# Acknowledging Uncertainty in Economic Forecasting. Some Insight from Confidence and Industrial Trend Surveys

**DOI:** 10.3390/e21040413

**Published:** 2019-04-18

**Authors:** Ana Jesús López-Menéndez, Rigoberto Pérez-Suárez

**Affiliations:** Department of Applied Economics, University of Oviedo, Campus del Cristo s/n, 33006 Oviedo, Asturias, Spain

**Keywords:** uncertainty, qualitative surveys, Shannon’s entropy, quadratic entropy, VAR, impulse-response analysis

## Abstract

The role of uncertainty has become increasingly important in economic forecasting, due to both theoretical and empirical reasons. Although the traditional practice consisted of reporting point predictions without specifying the attached probabilities, uncertainty about the prospects deserves increasing attention, and recent literature has tried to quantify the level of uncertainty perceived by different economic agents, also examining its effects and determinants. In this context, the present paper aims to analyze the uncertainty in economic forecasting, paying attention to qualitative perceptions from confidence and industrial trend surveys and making use of the related ex-ante probabilities. With this objective, two entropy-based measures (Shannon’s and quadratic entropy) are computed, providing significant evidence about the perceived level of uncertainty. Our empirical findings show that survey’s respondents are able to distinguish between current and prospective uncertainty and between general and personal uncertainty. Furthermore, we find that uncertainty negatively affects economic growth.

## 1. Introduction

In the context of a complex world characterized by high levels of uncertainty, several works have emphasized the need of acknowledging uncertainty in economic modeling and forecasting [1,2,3], also suggesting the convenience of complementing the predictions with the surrounding levels of uncertainty [4,5].

The controversial debate about the effects of uncertainty in consumers, managers, investors, …is not easy to solve due both to the lack of data and to methodological difficulties. Although the traditional practice consisted of reporting point predictions without specifying the attached probabilities, uncertainty about the prospects deserves increasing attention, and recent literature has tried to quantify the level of uncertainty perceived by different economic agents also examining its effects and determinants.

Within this context, the present paper aims to analyze the uncertainty around economic forecasts, paying attention to qualitative perceptions. With this purpose, the next section briefly describes the role of uncertainty in economic forecasting and the main difficulties that should be addressed in order to approach the level of uncertainty from surveys.

The materials and methods are presented in Section 3 where we set three different hypotheses referred to the measurement of forecasting uncertainty and its impact on economic growth. Since the estimation of uncertainty is closely related to the available information, this section also describes the statistical sources (barometers of the Spanish Center for Sociological Research and regional Industrial Trend Surveys) and the proposed measures (Shannon’s and quadratic entropy).

The empirical results are described in Section 4, where we summarize the main findings on the proposed hypotheses based on Confidence and Industrial Trend Surveys. Finally, section five contains the discussion of the obtained results and some concluding remarks.

## 2. Uncertainty in Economic Forecasting

In spite of the wide consensus on the main role of uncertainty in economic forecasting, it appears not to receive the academic attention it deserves, as emphasis is often made in best estimates and predictions without paying attention to the surrounding uncertainty. However, uncertainty has become increasingly important in economic forecasting due to both theoretical and empirical reasons and recent literature has tried to quantify the level of uncertainty perceived by different economic agents also examining its effects and determinants.

Different approaches can be used in the measurement of uncertainty, including statistical models and human judgement. While ex-post uncertainty has been usually studied by looking at forecasting errors, ex-ante uncertainty—which is particularly interesting from the economic point of view—could be estimated from survey data, as we intend in this work. With regard to the ex-post approach, empirical evidence including the M-competitions [6,7] shows that neither forecasting errors nor uncertainty are reduced with more sophisticated forecasting techniques or higher level of respondents’ expertise. From the ex-ante perspective, as explained by [8] the methodology is evolving with the types of surveys and datasets. Different proxies have been proposed to approach forecast uncertainty being one of the most popular disagreement, usually measured through the variance of the point forecasts. However, several authors [8,9,10] have emphasized the limitations of this approach, since disagreement between forecasters only considers the between component, and its reliability as a proxy for uncertainty will depend on several factors, as the stability and length of the forecasting horizon. In this context, the use of entropy-based measures seems to be a good option to take advantage of the information provided by forecasts surveys, including both the expected economic outcomes and the surrounding uncertainty levels. Unfortunately, as pointed out in [9] most of the professional surveys lack quantitative measures of uncertainty as they only aggregate the information of individuals’ assessment on the economic variables.

Measuring the level of uncertainty greatly depends on the information available to estimate probabilities that appear in uncertainty measures. A wide variety of existing surveys are summarized in Table 1, taking into account their size, level of expertise and information content.

The first category considered corresponds to surveys of professional forecasters (SPF), provided quarterly by the Federal Reserve Bank of Philadelphia, the European Central Bank and some other institutions, such as the Bank of England. Although the antecedents of SPF date from 1968 when the American Statistical Association and the National Bureau of Economic Research jointly started a quarterly survey of macroeconomic forecasters, the Federal Reserve Bank of Philadelphia assumed the responsibility for the survey and named it SPF in 1990. Similar investigations have been developed by the European Central Bank since 1999 (Survey of Professional Forecasters) and by the Bank of England since 1996 (Survey of External Forecasters). These highly specialized panels have an intermediate size (around 36 forecasters in the US-SPF and 75 forecasters in the EU-SPF) and collect forecasters’ expectations on key economic variables, such as inflation and GDP growth and unemployment rate, also including a particularly interesting feature: forecasters are asked to provide their subjective probabilities that a variable will fall into each of the predefined forecasting intervals, thus allowing the estimation of uncertainty from density forecasts as shown in [9,10]. With this aim, different approaches have been proposed to handle density functions, assuming some specific probability models such as the uniform [11], normal [12,13] or generalized beta [14].

Despite their success, surveys of professional forecasters also have some important limitations such as the difficulty of response and the lack of homogeneity, due to methodological changes and the replacement of forecasters.

The second category refers to panels of institutional or professional forecasters that are available for different countries, providing short-term predictions referred to the main economic aggregates (GDP and its components, employment, prices, etc.). These panels usually comprise a moderate number of recognized institutions including universities, research services of banks and economic analysis institutes. In the Spanish context, the private non-profit organization FUNCAS (a think tank dedicated to social and economic research, https://www.funcas.es). publishes the Spanish economy forecast panel, a survey carried out every two months among a panel of 19 institutions that has been studied in [15,16]. Although this kind of panel usually includes a consensus forecast (computed as the average) and some measures of dispersion (rank, variance, etc.) they do not allow the estimation of probabilities and uncertainty measures.

Expert elicitations are another interesting source of specialized information referring to future prospects and associated uncertainties, usually collected through subjective probabilities. This third category has been increasingly used in order to obtain experts judgments from scientists, engineers, and other analysts who are knowledgeable about particular issues and variables of interest, as described in [17] among others. Obviously, the size of these panels is quite small due to the required level of expertise and the difficulty of assigning the required probabilities.

Finally, the fourth category corresponds to confidence surveys, comprising a wide variety of initiatives performed for different countries and sectors, where a high number of economic agents (consumers, managers, etc.) show their positive or negative attitudes with regard to the current, previous or future economic activity. In the European framework, regular harmonized surveys are conducted for the member countries under the Joint Harmonized EU Program of Business and Consumer Surveys. The information provided by business and consumer confidence surveys has been proven to be extremely useful for short-term forecasting, detection of turning points and economic analysis [18,19]. Confidence survey data are generally presented as balances between the percentage of positive and negative answers to each question and their results are mainly used to compute synthetic indicators built on selected questions (confidence indicators, economic sentiment indicators, business climate indicators, etc.).

Furthermore, the vast amount of information provided by the participants in these surveys allows the estimation of frequentist probabilities and uncertainty measures, as we will show in the next sections of this paper.

## 3. Materials and Methods

Although the previously described surveys provide a huge amount of information, many empirical studies make exclusive use of consensus forecasts rather than analyzing individual forecasts and examining the surrounding level of uncertainty. Moreover, the estimation of uncertainty has mainly been based on subjective probabilities provided by the surveys of professional forecasters or the experts’ elicitations, while this approach has scarcely been used in the case of confidence surveys. In this paper we aimed to fill this gap, approaching the economic uncertainty with probabilities estimated from confidence and industrial trend surveys. More specifically, we focused on the barometers developed by the Spanish Center for Sociological Research (CIS) and the regional Industrial trend Surveys (ECI), referred to as Asturias, providing significant evidence about both the economic situation and the encompassing uncertainty.

### 3.1. Hypotheses

Three hypotheses have been proposed referred to the informational content of the considered surveys and the relationship between uncertainty and economic growth:

1. Confidence surveys allow an adequate estimate of the economic situation and the surrounding uncertainty.
2. A survey’s respondents can properly distinguish between current and prospective uncertainty and between general and personal uncertainty.
3. Uncertainty negatively affects economic growth.

With the aim of testing the proposed hypotheses we firstly describe the available information, respectively provided by the barometer of the Spanish Center of Sociological Research and the regional Industrial Trend Survey. Besides supplying synthetic indicators, both sources allow the estimation of probabilities and uncertainty levels through entropy-based measures. More specifically in this paper we used Shannon’s and quadratic Indexes, thus allowing a comparison of the uncertainty levels estimated by both expressions.

Furthermore, the estimation of econometric models allows a more detailed analysis about the causal relationship and the impact of uncertainty on economic growth. Thus, vector autoregresive (VAR) models were estimated, and their results are described in Section 4.

### 3.2. Data Description: Confidence Barometers and Industrial Trend Surveys

CIS is an independent entity assigned to the Ministry of the Presidency, and gathers the necessary data for research in very different fields, carrying out a wide variety of surveys, whose data is in the public domain. The CIS databank includes confidence barometers, polls carried out since 1994 on a monthly basis (except in August), with the aim of measuring Spanish public opinion. As described in the CIS website [20] these polls involve interviews with around 2500 randomly-chosen people from all over the country, including a block of variable questions which focuses on the assessment of both the economic situation in Spain and the personal economic situation, as described in Table 2.

Microdata provided by the monthly polls can be downloaded from the CIS website www.cis.es and allow the calculation of probabilities based on relative frequencies assigned to the alternative options.

Regarding the Spanish industrial trend surveys, the Ministry of Industry, Trade and Tourism, and also some regional statistical offices develop qualitative surveys with the aim of catching the opinion of industrial managers about the current situation and future prospects. More specifically, the questionnaire is directed to the management industrial personnel and compiles qualitative information referred to the present levels of the portfolio orders and the production, sale prices and employment expected for the next months.

Three alternative answers (high, normal or low) are provided for those questions reflecting the present level, while the options to increase, to stay or to diminish can be selected if the questions refer to the expected tendency. The individual answers given to the different questions are aggregated in order to obtain series by classes and categories and the balance between the extreme options provides an indicator with values oscillating between +100 and −100 (totally‘ optimistic and pessimistic situations). The results for each variable can also be summarized through the industrial climate indicator (ICI) computed as an arithmetic mean of the balances of the portfolio orders, the production expectations and, with the opposite sign, the level of finished product stocks. This composite indicator is widely used to provide a global vision of the industrial confidence in relation to the conjunctural evolution. In fact, as the leading indicator signals summarized in the ICI are assumed to happen before the economy turning points, this index can be used as a leading indicator of economic activity allowing the obtention of economic turning point forecasts as shown in [16].

Since the estimation of uncertainty requires detailed information about individuals perceptions we focus on the regional industrial trend survey referred to Asturias, whose databank is fully available from [21] allowing the estimation of the corresponding probabilities.

### 3.3. Shannon’s and Quadratic Entropy Measures

Although qualitative surveys have been extensively used to obtain synthetic indicators, few attempts have been made in order to quantify the uncertainty level perceived by the respondents. In this paper we aim at filling this gap, and also analyzing to which extent the level of uncertainty perceived by the experts is related with the economic situation.

Entropy measures provide a suitable framework for our goal, as entropy is a function of the probability distribution and not a function of the actual values taken by the random variable. Since microdata of qualitative surveys allow the estimation of the probabilities assigned to each possible outcome, entropy measures can also be estimated. Thus, given the set of *n* distinct mutually exclusive options for a specific question, the individual responses allow the estimation of frequency probabilities $p_{i},\forall i=1,\ldots n$ such that $p_{i}\geq 0,\sum_{i}p_{i}=1$. Shannon [22] defines the information content of a single outcome as $h\left(p_{i}\right)=\log\left(\frac{1}{p_{i}}\right)$. According to this definition, observing a rare event provides much more information than observing another, more probable outcome.

In this context, Shannon’s entropy is defined as the expected amount of information and can be computed as $H=-\sum_{i}p_{i}\log\left(p_{i}\right)$. This expression plays a central role since it fulfills a number of interesting properties which, as shown in [22] substantiate it as a reasonable measure of information, choice or uncertainty:

1. H=0 if and only if all the $p_{i}$ but one are zero, this one having the value unity. Thus the result of *H* is null only when we are certain about the outcome, and otherwise *H* is positive.
2. For a given *n*, *H* is a maximum and equal to log(n) when all the $p_{i}$ are equal $p_{i}=\frac{1}{n},\forall i=1,2,\ldots ,n$. This is also intuitively the most uncertain situation.
3. Any change toward equalization of the probabilities $p_{1},p_{2},\ldots ,p_{n}$ increases *H*. Thus, if $p_{1}<p_{2}$ and we increase $p_{1}$ decreasing $p_{2}$ an equal amount so that $p_{1}$ and $p_{2}$ are more nearly equal, then *H* increases. More generally, if we perform any averaging operation on the $p_{i}$ of the form $p_{i}^{{}^{\prime }}=\sum_{j}a_{ij}p_{j}$ where $\sum_{i}a_{ij}=\sum_{j}a_{ij}=1$ and $a_{ij}\geq 0,\forall i,j=1,\ldots ,n$ then *H* increases, except in the case where this transformation amounts to no more than a permutation of the $p_{i}$ with *H* remaining the same.

Following a similar approach, Pérez [23] proposes the individual quadratic entropy, which can be computed for a single outcome as $h^{2}\left(p_{i}\right)=2\left(1-p_{i}\right)$. According to this proposal, the quadratic entropy is quantified as twice the distance of the probability of an event from the true outcome, and similarly to Shannon’s measure, the information provided by a rare event is higher than the information corresponding to a more likely one.

Given a set of probabilities $p_{i},\forall i=1,\ldots ,n$ such that $p_{i}\geq 0,\sum_{i}p_{i}=1$, the quadratic entropy is defined in [23] as the expected value of the individual quadratic entropies, given by the expression $H^{2}=2\sum_{i}p_{i}\left(1-p_{i}\right)$. This is a suitable measure of uncertainty since it fulfils the requirements proposed by Shannon. More specifically:

1. $H^{2}=0$ if and only if all the $p_{i}$ but one are zero, this one having the value unity.
2. For a given *n*, $H^{2}$ is a maximum when all the $p_{i}$ are equal $p_{i}=\frac{1}{n},\forall i=1,2,\ldots ,n$. This maximum value, corresponding to the most uncertain situation, is given by the expression $2(1-\frac{1}{n})$ and in the limit it takes a value of two.
3. Any change toward equalization of the probabilities $p_{1},p_{2},\ldots ,p_{n}$ increases the quadratic entropy $H^{2}$. Thus, if we perform any averaging operation on the $p_{i}$ of the form $p_{i}^{{}^{\prime }}=\sum_{j}a_{ij}p_{j}$ where $\sum_{i}a_{ij}=\sum_{j}a_{ij}=1$ and $a_{ij}\geq 0,\forall i,j=1,\ldots ,n$ then $H^{2}$ increases, except if this transformation is only a permutation of the $p_{i}$ (in this case $H^{2}$ does not change, since the quadratic entropy fulfils the property of symmetry).

The quadratic measure has been successfully used in different economic applications, including the evaluation of forecasts [24,25]. Taking into account its suitable behavior, in this paper we propose the joint use of Shannon’s and quadratic entropy to approach the level of uncertainty.

## 4. Results

This section summarizes the results obtained from the CIS barometer and the industrial confidence survey, providing empirical evidence referred to the three proposed hypotheses. As previously described, the available information allows us to compute uncertainty levels through Shannon’s and quadratic entropy measures, respectively given by the expressions:

(1) $$H=-\sum_{i=1}^{n}p_{i}\log p_{i}$$

(2) $$H^{2}=2\sum_{i=1}^{n}p_{i}\left(1-p_{i}\right).$$

As these expressions verify the reasonable properties to be considered as suitable measures of uncertainty they have been used in a complementary way.

### 4.1. Hypothesis 1

**Figure 1:**
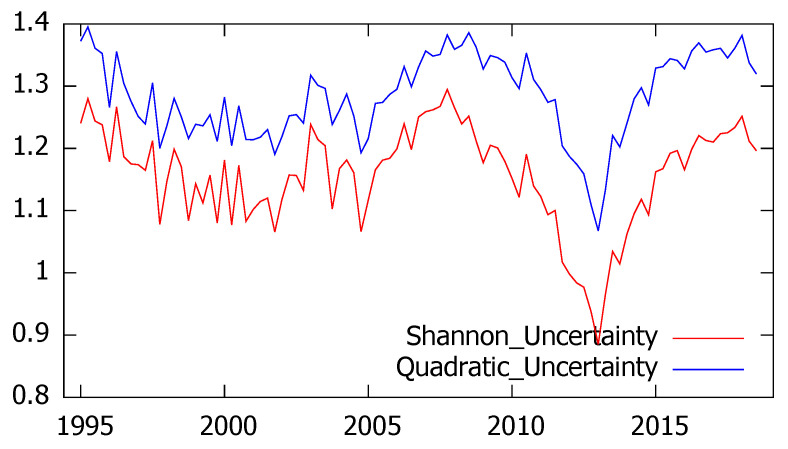
Evolution of Shannon’s and quadratic uncertainty associated to current economic situation in Spain.

According to the first proposed hypothesis, confidence surveys allow an adequate estimate of the economic situation and the surrounding uncertainty. With the aim of testing this assumption we first consider the CIS Confidence barometers collecting extremely interesting information referred to respondents’ perception about both the economic situation in Spain and their personal situation. Since the CIS survey is not available in august, we have used quarterly series. The results of both entropy measures are represented in Figure 1, showing a very similar evolution. As expected, Shannon’s and quadratic entropy appear to be highly correlated (the linear correlation coefficient between them reaches the value 0.91) and the level of uncertainty significantly increases between 2005 and 2007 according to both measures. Subsequently, since the end of 2007, a decreasing pattern is observed until the first quarter of 2013 when both indicators reach their minimum value and the uncertainty starts a new rise.

The analysis of these time series confirms that seasonality does not affect the levels of perceived uncertainty (the Kruskal–Wallis test fails to reject the null hypothesis of non seasonality and the same conclusion is obtained through an OLS regression with periodic dummy variables, that are found to be non-significant). It is also interesting to remark that the “herding effect” which has been largely studied in panels of forecasters does not appear in this case, as the respondents have been randomly selected and there is no influence among them.

A similar analysis has been performed on the industrial trend survey that, as we have previously described, aims at catching industrial managers’ opinions about the present and future economic situation. In this case we analyze the information referred to the region of Asturias from January 1990 to December 2018 and, although the questionnaire includes qualitative information related to several variables, we mainly focus on industrial production.

**Figure 2:**
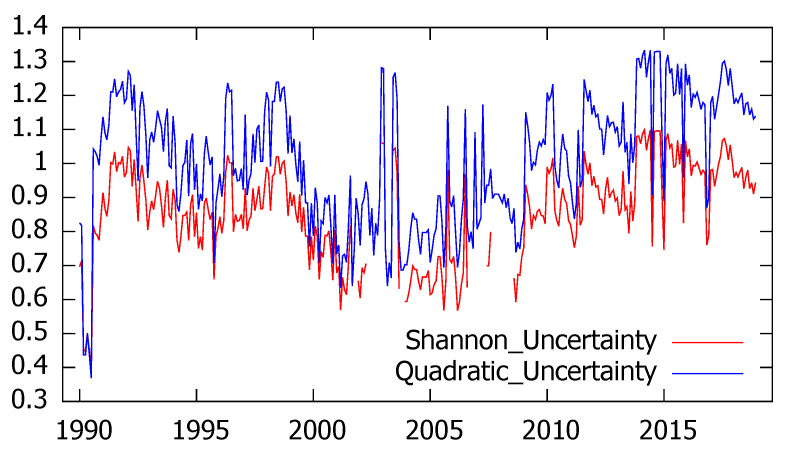
Evolution of Shannon’s and quadratic uncertainty associated to current industrial production in Asturias.

Experts’ answers were used to compute the probabilities associated to the three alternative options for the current output level (high, normal and low), leading to the estimation of monthly series for Shannon and quadratic uncertainty whose results are plot in Figure 2.

As expected, both entropy measures provide quite similar results when measuring uncertainty referred to the current industrial production, leading to a linear correlation coefficient of 0.98.

### 4.2. Hypothesis 2

**Figure 3:**
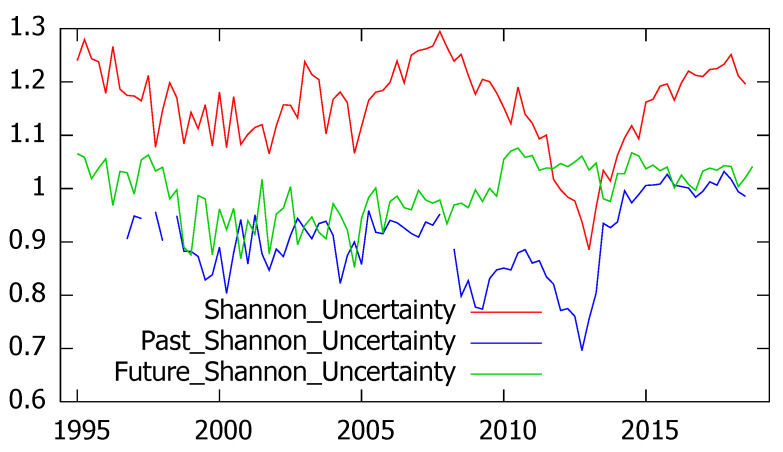
Shannon’s uncertainty for current, retrospective and prospective economic situation in Spain.

**Figure 4:**
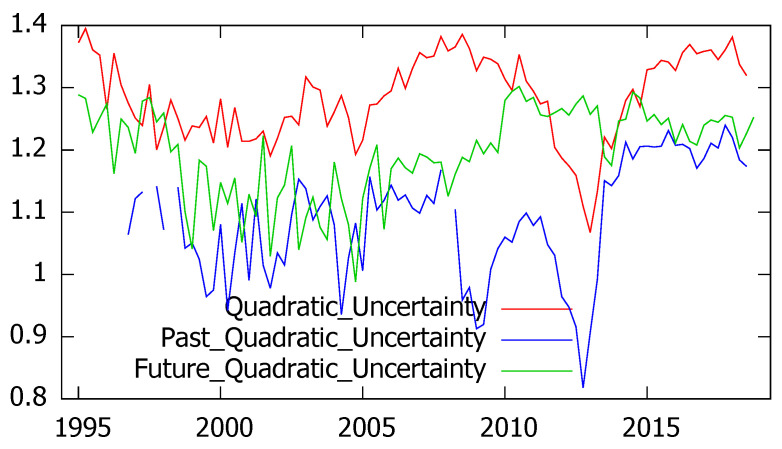
Quadratic uncertainty for current, retrospective and prospective economic situation in Spain.

The second hypothesis refers to the ability of survey’s respondents to distinguish between current and prospective uncertainty and between general and personal uncertainty. Since the CIS barometers include current, retrospective and prospective assessments of the economic situation in Spain, we have compared the corresponding levels of Shannon’s and quadratic uncertainty, represented in Figure 3 and Figure 4. As it can be seen, according to both entropy measures current uncertainty is found to be higher than prospective uncertainty, which generally exceeds past uncertainty. However, some exceptions are found, corresponding to years 2012 and 2013 when the present uncertainty reaches its minimum values and is exceeded by prospective uncertainty.

As we have seen in the previous figures, Shannon’s and quadratic entropy mostly agree in the quantification of uncertainty. No matter if we consider the general or the personal situation or if uncertainty refers to present, past or future periods, the correlation coefficients always exceed 90% as summarized in Table 3.

**Figure 5:**
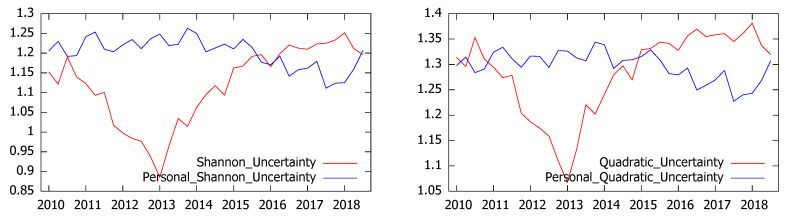
Shannon’s (**left**) and quadratic (**right**) uncertainty for personal and Spanish economic situation.

In order to analyze to which extent survey’s respondents can properly distinguish between general and personal uncertainty we have also studied the perceptions about their personal economic situation. Although these series, represented in Figure 5 were quite short (they are only available since 2010) and therefore should be considered cautiously, the results show that until 2015 the level of uncertainty was higher when it refers to the personal situation. However, the perception of personal uncertainty seems to be more stable than that referred to the general economic situation and both measures are negatively correlated (−0.73 and −0.6 for Shannon and quadratic uncertainty respectively).

It is also interesting to mention that this situation changes when we focus on uncertainty about the future. In this case, we find no significant correlation between personal and general uncertainties, measured either with Shannon or quadratic entropy.

Regarding the relationship between current and prospective uncertainty, the findings differ from personal to country’s uncertainty (Table 4). It is interesting to remark that—independently of the measure of entropy used—when we pay attention to the personal situation there is a strong relationship between current and prospective uncertainty while this correlation does not exist when we focus on the assessment of the general economic situation. These findings confirm that the respondents were able to properly distinguish the perceptions related to their own economic situation and prospects from those referred to the country as a whole.

With regard to the industrial trend surveys, the experts’ answers referred to future prospects (whose alternative options are to increase, to stay and to decrease) allow the estimation of future uncertainty, leading to similar results for Shannon’s and quadratic entropy (the correlation coefficient amounted to 0.99). As in the previous application, the obtained results show that the respondents clearly distinguished between present and prospective uncertainty. In fact, regardless of the entropy measure considered, uncertainty referred to the present industrial output is found to be higher and more stable than uncertainty referred to the future industrial production.

**Figure 6:**
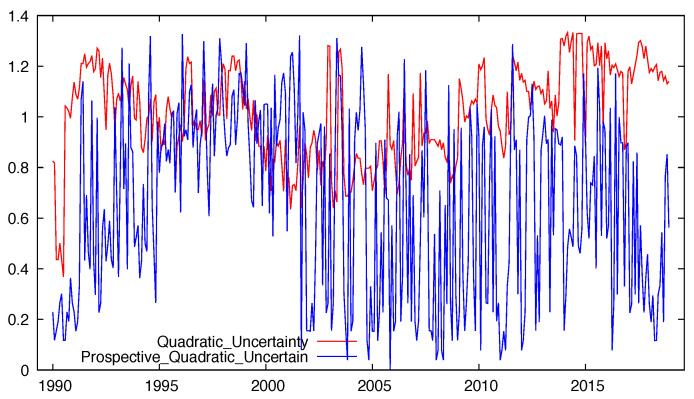
Evolution of quadratic uncertainty associated to current and prospective industrial production in Asturias.

These findings, represented in Figure 6 for the quadratic entropy, have been corroborated through paired difference tests, leading to the conclusion that the expected current uncertainty significantly exceeds prospective uncertainty.

### 4.3. Hypothesis 3

According to the third hypothesis, which we consider especially interesting, uncertainty negatively affects economic growth. In order to analyze this assumption we first focus on the CIS barometer, considering the estimated Shannon’s and quadratic entropy together with two additional quarterly series: the annual GDP growth rate and a synthetic indicator.

Denoting by $X_{t}$ the quarterly GDP, the related annualized growth rate is given by the expression $g=\frac{X_{t}}{X_{t-4}}-1$.

Furthermore, following a widely extended practice in this kind of surveys, a synthetic index can be computed in order to summarize the answers. Focusing on the assessment of the current economic situation in Spain, this indicator can be easily obtained as follows: $SI=2p_{very\_good}+p_{good}-p_{bad}-2p_{very\_bad}$, where $p_{very\_good},\;p_{good},\;p_{bad},\;p_{very\_bad}$ represent the probabilities assigned to each of the considered categories, estimated through the corresponding relative frequencies.

**Figure 7:**
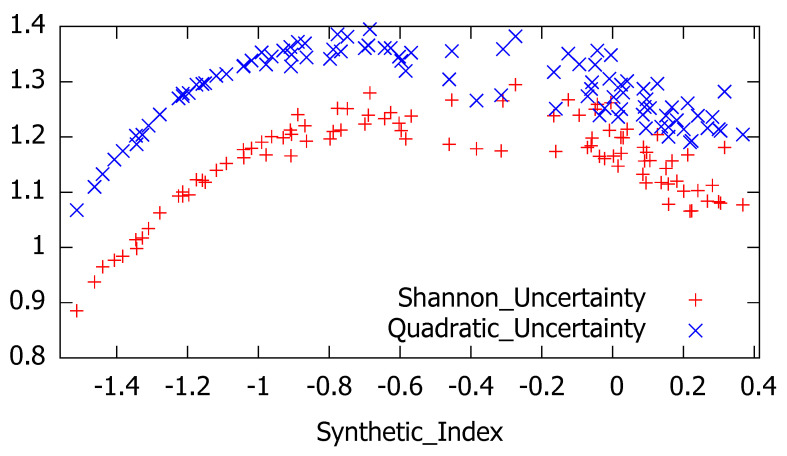
Shannon’s and quadratic uncertainty versus synthetic index.

Once this indicator has been computed we can analyze the relationship between the perceived economic situation and the corresponding level of uncertainty. Although these quarterly series appear to be contemporaneously uncorrelated, the scatter diagram represented in Figure 7 provides some interesting hints about the parabolic pattern of uncertainty regarding the synthetic index.

As it can be seen in this graph, low uncertainty with low dispersion is associated with very negative perceptions of the economic situation, whilst as perceptions of economic situation increase, so too do measures of uncertainty with associated increasing dispersion.

With the aim of examining how uncertainty impacts on economic activity, a more detailed analysis has been developed through VAR models. More specifically, we propose VAR models involving the economic growth, the synthetic index and the uncertainty measure, and we run two versions by using either Shannon’s entropy or quadratic entropy as the measure of uncertainty. We estimated both VAR models on quarterly data from 1996 to 2018 (T = 89) and, following the commonly used information criteria (Akaike, Schwartz and Hannah–Quinn), we considered two lags (*p* = 2). Table A1 and Table A2 in the Appendix A collect the VAR estimation results.

It is interesting to notice that the Granger causality test (whose null hypothesis is “no Granger causality”) leads to the *p*-values collected in Table 5 and Table 6, showing that variations in GDP are explained by both the synthetic index and the level of uncertainty, regardless of the entropy measure used. Moreover, uncertainty was found to Granger cause the synthetic index at the 10% level.

**Figure 8:**
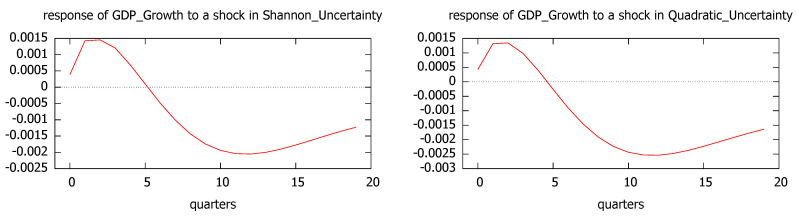
Impulse responses of GDP growth to a shock in Shannon’s uncertainty (**left**, VAR 1) and quadratic uncertainty (**right**, VAR 2).

**Figure 9:**
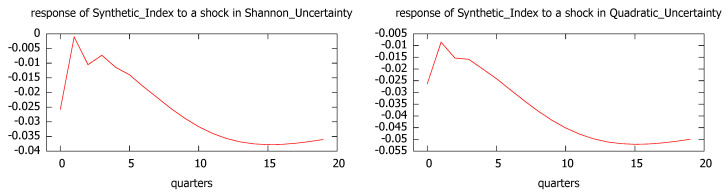
Impulse responses of the synthetic index to a shock in Shannon’s uncertainty (**left**, VAR 1) and quadratic uncertainty (**right**, VAR 2).

Since uncertainty causes economic growth, we have also analyzed the impulse responses for GDP growth and the synthetic index to a one standard deviation shock in the uncertainty level, measured both by Shannon and quadratic entropy. The results are plot in Figure 8 and Figure 9, showing that the effects of one standard deviation shock to the uncertainty in economic growth are mostly negative with their largest impacts around 12–15 months.

According to the impulse-response analysis, the behavior is quite robust with regard both to the economic indicator (GDP growth and synthetic index) and the uncertainty measure (Shannon’s and quadratic entropy).

**Figure 10:**
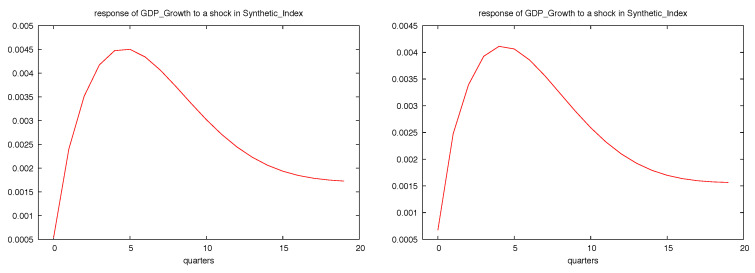
Impulse response of GDP growth to a shock in the synthetic index according to VAR 1 (**left**) and VAR2 (**right**).

Regarding the impact of the synthetic index on GDP growth, Figure 10 represents the impulse-response analysis for one standard deviation shock in the synthetic index. As expected, the response in this case is positive and faster, with its largest impact taking place around five months.

Following the same method we examine the relationship between uncertainty and industrial production. As in the previous analysis we estimate two VAR models including, in this case, four monthly series, corresponding to the regional IPI, the ICI, the synthetic index (SI) and the level of uncertainty.

These series have been obtained from SADEI [21], the regional statistical office of Asturias which provides monthly information about the industrial production index (currently referred to year 2010) and the ICI, a leading indicator of economic activity [16] computed as an arithmetic mean of the balances of the portfolio orders, the production expectations and—with the opposite sign—the level of stocks. Regarding the Synthetic Index, it has been computed as in the previous subsection from the balance of positive and negative answers referred to industrial output, using the estimated frequency probabilities.

Finally, with regard to the level of uncertainty, two VAR models have been estimated, using Shannon’s entropy in the first one and quadratic entropy in the second. Since Shannon’s index cannot be computed for some months with null probability in any of the categories we have restricted the sample size in both models (T = 124) in order to provide fully homogeneous results.

It is interesting to remark that, taking into account the series analyzed, VAR specification includes in this case constant, trend and seasonality. Following the information criteria, only one lag was considered.

**Figure 11:**
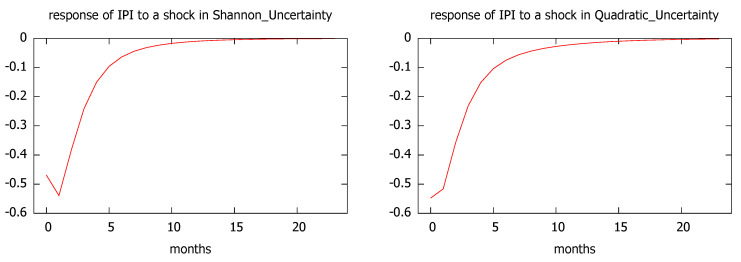
Impulse response of industrial production index (IPI) to a shock in Shannon’s (**left**) and quadratic uncertainty (**right**).

The estimation results are collected in the Appendix A (Table A3 and Table A4) and the conclusions show outstanding similarities for the two uncertainty measures, as it can be seen in Figure 11. As expected, the impulse responses of the regional industrial production index to a one standard deviation shock to the uncertainty level are negative with their largest impacts during the first two periods and a quick recovery in the medium run.

## 5. Discussion and Concluding Remarks

Our empirical results show that qualitative surveys can be successfully used to approach both the economic situation and the surrounding uncertainty, thus agreeing with the first proposed hypothesis. More specifically, the information provided by the respondents to the CIS barometer and the industrial trend survey confirms the usefulness of both sources and the adequacy of entropy-based measures to approach uncertainty. In addition, we find that—as indicated by previous works [2]—the level of expertise does not affect the adequacy of respondents’ answers.

According to the two empirical applications, based in confidence barometers and industrial trend surveys, Shannon’s and quadratic entropy mostly agree in the quantification of uncertainty, no matter if we consider the Spanish or the personal economic situation or if uncertainty refers to present, past or future periods.

Regarding the second hypothesis, the available information suggests that surveys respondents can properly distinguish between current and prospective uncertainty and between general and personal uncertainty. According to the CIS barometer and the Industrial Trend Survey, current uncertainty is higher than prospective uncertainty, regardless of the measure used. Furthermore, the CIS barometer provides significant evidence about the capability of survey’s respondents to distinguish between personal and national uncertainty: first, the perception of personal uncertainty seems to be more stable than that referred to the Spanish economic situation and second, a strong positive correlation is found between current and prospective uncertainty referred to the personal situation, unlike what happens when we focus on the economic situation of the country. Finally, our empirical applications show that uncertainty negatively affects economic growth, providing evidence about the responses of economic growth and industrial production to a shock in the uncertainty measures.

The estimation of VAR models leads to some interesting findings that broadly match with previous works as [26,27,28]. More specifically, the adverse impacts of uncertainty shocks on economic activity have been documented among others in [26,27] while [28] provides significant evidence about the fall of industrial production as a response to a volatility shock.

Our results based on the CIS barometer are quite robust, since they confirm that uncertainty shocks, regardless of the entropy measure used, have a negative impact on economic activity, whether measured through GDP growth or the synthetic index. According to the impulse-response analysis, the largest impacts take place around 12–15 months, followed by a slow recovery. Similarly, when we focus on the industrial trend survey, we find that one standard deviation shock to the uncertainty level (measured either by Shannon’s or quadratic entropy) leads to sharp reductions in the regional industrial production, with a quick recovery in the medium run.

Despite their limitations, these interesting findings confirm the potential of qualitative surveys in the assessment of economic uncertainty also suggesting the need of further research in this field.

## Figures and Tables

**Table 1:** Main typologies of forecasting surveys.

Survey	Size	Level of Expertise	Information
Surveys of professional forecasters	Medium	High	Detailed (Density forecasts)
Panels of professional forecasters	Medium	High	Reduced (consensus forecasts)
Expert elicitations	Small	Very high	Detailed (subjective probabilities)
Confidence surveys	High	Low/Medium	Medium (frequency probabilities)

**Table 2:** Spanish Center for Sociological Research (CIS) confidence barometer.

Items	Options
Assessment of the current economic situation in Spain	Very Good, Good, Intermediate, Bad, Very Bad
Retrospective assessment of the economic situation in Spain (one year before)	Better. Equal, Worse
Prospective assessment of the economic situation in Spain (one year)	Better. Equal, Worse
Assessment of the current personal economic situation	Very Good, Good, Intermediate, Bad, Very Bad
Prospective assessment of the personal economic situation (one year)	Better, Equal, Worse

**Table 3:** Correlation coefficients between Shannon’s and quadratic Uncertainty.

	Spanish Economy	Personal Economy
Current	0.91	0.97
Retrospective (one year before)	0.99	—
Prospective (one year)	0.99	0.97

**Table 4:** Correlation coefficients between current and prospective uncertainty.

	Spanish Economy	Personal Economy
Shannon’s Entropy	−0.023	0.816
Quadratic Entropy	0.19	0.769

**Table 5:** *p*-values for the Granger causality tests (F-test of zero restrictions) in vector autoregresive (VAR) 1.

	GDP Growth	Synthetic Index	Shannon Uncertainty
All lags of GDP growth	0.0000	0.0633	0.1369
All lags of synthetic index	0.0059	0.0000	0.6183
All lags of Shannon’s uncertainty	0.0059	0.1200	0.0000

**Table 6:** *p*-values for the Granger causality tests (F-test of zero restrictions) in VAR 2.

	GDP Growth	Synthetic Index	Quadratic Uncertainty
All lags of GDP growth	0.0000	0.0510	0.1571
All lags of synthetic index	0.0031	0.0000	0.5184
All lags of quadratic uncertainty	0.0269	0.2874	0.0000

## Appendix A

**Table A1:** Results of VAR estimation for GDP growth, synthetic index and Shannon’s uncertainty.

	Equation (1): GDp Growth	Equation (2): Synthetic Index	Equation (3): Shannon’s Uncertainty
const	$\underset{\left(0.0077\right)}{0.0075}$	$\underset{\left(0.1992\right)}{0.0417}$	$\underset{\left(0.0784\right)}{0.1898{\;}^{**}}$
GDP_Growth_1	$\underset{\left(0.0792\right)}{1.4824{\;}^{***}}$	$\underset{\left(2.0422\right)}{4.6636{\;}^{**}}$	$\underset{\left(0.8033\right)}{1.0144}$
GDP_Growth_2	$\underset{\left(0.0826\right)}{-0.6090{\;}^{***}}$	$\underset{\left(2.1275\right)}{-3.7670{\;}^{*}}$	$\underset{\left(0.8369\right)}{-0.3986}$
Synthetic_Index_1	$\underset{\left(0.0044\right)}{0.0159{\;}^{***}}$	$\underset{\left(0.1129\right)}{0.8687{\;}^{***}}$	$\underset{\left(0.0444\right)}{0.0225}$
Synthetic_Index_2	$\underset{\left(0.0043\right)}{-0.0123{\;}^{***}}$	$\underset{\left(0.1098\right)}{0.0896}$	$\underset{\left(0.0431\right)}{-0.0319}$
Shannon_Uncertainty_1	$\underset{\left(0.0105\right)}{0.0305{\;}^{***}}$	$\underset{\left(0.2713\right)}{0.4737{\;}^{*}}$	$\underset{\left(0.1067\right)}{0.5175{\;}^{***}}$
Shannon_Uncertainty_2	$\underset{\left(0.0102\right)}{-0.0332{\;}^{***}}$	$\underset{\left(0.2626\right)}{-0.5452{\;}^{**}}$	$\underset{\left(0.1033\right)}{0.3018{\;}^{***}}$

Note: Standard deviation in parenthesis; ∗ p<0.1,∗∗ p<0.05,∗∗∗ p<0.01.

**Table A2:** Results of VAR estimation for GDP growth, synthetic index and quadratic uncertainty.

	Equation (1): GDP Growth	Equation (2): Synthetic Index	Equation (3): Quadratic Uncertainty
const	$\underset{\left(0.0099\right)}{0.0101}$	$\underset{\left(0.2523\right)}{0.1756}$	$\underset{\left(0.0797\right)}{0.1781{\;}^{**}}$
GDP_Growth_1	$\underset{\left(0.0810\right)}{1.4832{\;}^{***}}$	$\underset{\left(2.0709\right)}{4.7214{\;}^{**}}$	$\underset{\left(0.6543\right)}{0.8938}$
GDP_Growth_2	$\underset{\left(0.0848\right)}{-0.6036{\;}^{***}}$	$\underset{\left(2.1687\right)}{-3.5476}$	$\underset{\left(0.6852\right)}{-0.4385}$
Synthetic_Index_1	$\underset{\left(0.0044\right)}{0.0143{\;}^{***}}$	$\underset{\left(0.1132\right)}{0.8280{\;}^{***}}$	$\underset{\left(0.0358\right)}{-0.0048}$
Synthetic_Index_2	$\underset{\left(0.0043\right)}{-0.0111{\;}^{**}}$	$\underset{\left(0.1093\right)}{0.1169}$	$\underset{\left(0.0345\right)}{-0.0086}$
Shannon_Uncertainty_1	$\underset{\left(0.0134\right)}{0.0315{\;}^{**}}$	$\underset{\left(0.3447\right)}{0.3344}$	$\underset{\left(0.1089\right)}{0.6423{\;}^{***}}$
Shannon_Uncertainty_2	$\underset{\left(0.0132\right)}{-0.0362{\;}^{***}}$	$\underset{\left(0.3375\right)}{-0.5131}$	$\underset{\left(0.1066\right)}{0.2058{\;}^{*}}$

Note: Standard deviation in parenthesis; ∗ p<0.1,∗∗ p<0.05,∗∗∗ p<0.01.

**Table A3:** Results of VAR estimation for IPI, industrial climate indicator (ICI), synthetic index and Shannon’s uncertainty.

	Equation (1): IPI	Equation (2): ICI	Equation (3): Synthetic Index	Equation (4): Shannon’s Uncertainty
const	$\underset{\left(13.5687\right)}{78.2967{\;}^{***}}$	$\underset{\left(21.9591\right)}{-7.7117}$	$\underset{\left(30.3293\right)}{-50.1833}$	$\underset{\left(0.2651\right)}{0.4149}$
IPI_1	$\underset{\left(0.0892\right)}{0.4264{\;}^{***}}$	$\underset{\left(0.1444\right)}{-0.2030}$	$\underset{\left(0.1995\right)}{0.3989{\;}^{**}}$	$\underset{\left(0.0017\right)}{-0.0008}$
ICI_1	$\underset{\left(0.0539\right)}{0.0655}$	$\underset{\left(0.0872\right)}{0.5432{\;}^{***}}$	$\underset{\left(0.1204\right)}{0.5030{\;}^{***}}$	$\underset{\left(0.0011\right)}{0.0023{\;}^{**}}$
Synthetic_Index_1	$\underset{\left(0.0221\right)}{0.0253}$	$\underset{\left(0.0358\right)}{0.0512}$	$\underset{\left(0.0495\right)}{0.6958{\;}^{***}}$	$\underset{\left(0.0004\right)}{-0.0010{\;}^{**}}$
Shannon_Uncertainty_1	$\underset{\left(4.0951\right)}{-5.2332}$	$\underset{\left(6.6274\right)}{3.3735}$	$\underset{\left(9.1536\right)}{-14.5058}$	$\underset{\left(0.0800\right)}{0.5159{\;}^{***}}$
S1	$\underset{\left(1.7703\right)}{5.6129{\;}^{***}}$	$\underset{\left(2.8650\right)}{-5.9058{\;}^{**}}$	$\underset{\left(3.9570\right)}{4.8208}$	$\underset{\left(0.0346\right)}{-0.0036}$
S2	$\underset{\left(1.6913\right)}{2.1790}$	$\underset{\left(2.7372\right)}{-8.3030{\;}^{***}}$	$\underset{\left(3.7805\right)}{1.7326}$	$\underset{\left(0.0330\right)}{0.0462}$
S3	$\underset{\left(1.7113\right)}{9.2197{\;}^{***}}$	$\underset{\left(2.7695\right)}{-6.2971{\;}^{**}}$	$\underset{\left(3.8251\right)}{2.6542}$	$\underset{\left(0.0334\right)}{0.0175}$
S4	$\underset{\left(1.7058\right)}{-0.6028}$	$\underset{\left(2.7606\right)}{-5.3639{\;}^{*}}$	$\underset{\left(3.8129\right)}{2.9481}$	$\underset{\left(0.0333\right)}{0.0333}$
S5	$\underset{\left(1.7316\right)}{7.2989{\;}^{***}}$	$\underset{\left(2.8024\right)}{-11.0793{\;}^{***}}$	$\underset{\left(3.8706\right)}{3.8561}$	$\underset{\left(0.0338\right)}{0.0151}$
S6	$\underset{\left(1.7026\right)}{4.0416{\;}^{**}}$	$\underset{\left(2.7554\right)}{-7.6083{\;}^{***}}$	$\underset{\left(3.8057\right)}{6.5073{\;}^{*}}$	$\underset{\left(0.0332\right)}{0.0112}$
S7	$\underset{\left(1.7073\right)}{3.1868{\;}^{*}}$	$\underset{\left(2.7631\right)}{-9.1271{\;}^{***}}$	$\underset{\left(3.8163\right)}{3.4087}$	$\underset{\left(0.0333\right)}{-0.0083}$
S8	$\underset{\left(1.7324\right)}{-7.7571{\;}^{**}}$	$\underset{\left(2.8037\right)}{1.3488}$	$\underset{\left(3.8724\right)}{1.8777}$	$\underset{\left(0.0338\right)}{0.0934{\;}^{***}}$
S9	$\underset{\left(2.0608\right)}{8.9359{\;}^{***}}$	$\underset{\left(3.3351\right)}{-12.5681{\;}^{***}}$	$\underset{\left(4.6064\right)}{6.5238}$	$\underset{\left(0.0403\right)}{-0.0287}$
S10	$\underset{\left(1.6487\right)}{8.6702{\;}^{***}}$	$\underset{\left(2.6682\right)}{-5.2552{\;}^{*}}$	$\underset{\left(3.6852\right)}{4.2867}$	$\underset{\left(0.0322\right)}{0.0222}$
S11	$\underset{\left(1.6597\right)}{3.7736{\;}^{**}}$	$\underset{\left(2.6860\right)}{-5.0736{\;}^{*}}$	$\underset{\left(3.7099\right)}{1.2455}$	$\underset{\left(0.0324\right)}{-0.0004}$
time	$\underset{\left(0.0230\right)}{-0.0860{\;}^{***}}$	$\underset{\left(0.0372\right)}{0.1221{\;}^{***}}$	$\underset{\left(0.0514\right)}{0.0567}$	$\underset{\left(0.0004\right)}{0.0002}$

Note: Standard deviation in parenthesis; ∗ p<0.1,∗∗ p<0.05,∗∗∗ p<0.01.

**Table A4:** Results of VAR estimation for IPI, ICI, synthetic index and quadratic uncertainty.

	Equation (1): IPI	Equation (2): ICI	Equation (3): Synthetic Index	Equation (4): Quadratic Uncertainty
const	$\underset{\left(5.0065\right)}{11.9661{\;}^{**}}$	$\underset{\left(8.1289\right)}{28.2318{\;}^{***}}$	$\underset{\left(12.0858\right)}{-34.1409{\;}^{***}}$	$\underset{\left(0.1196\right)}{0.6871{\;}^{***}}$
IPI_1	$\underset{\left(0.0357\right)}{0.8428{\;}^{***}}$	$\underset{\left(0.0580\right)}{-0.1463{\;}^{**}}$	$\underset{\left(0.0861\right)}{0.2226{\;}^{**}}$	$\underset{\left(0.0009\right)}{-0.0032{\;}^{***}}$
ICI_1	$\underset{\left(0.0201\right)}{-0.0067}$	$\underset{\left(0.0326\right)}{0.8077{\;}^{***}}$	$\underset{\left(0.0485\right)}{0.1322{\;}^{***}}$	$\underset{\left(0.0005\right)}{0.0011{\;}^{**}}$
Synthetic_Index_1	$\underset{\left(0.0130\right)}{0.0130}$	$\underset{\left(0.0211\right)}{0.0231}$	$\underset{\left(0.0314\right)}{0.8162{\;}^{**}}$	$\underset{\left((0.0003)\right)}{-0.0002}$
Quadratic_Uncertainty_1	$\underset{\left(1.8431\right)}{-2.2750}$	$\underset{\left(2.9924\right)}{2.3620}$	$\underset{\left(4.4491)\right)}{-4.8080}$	$\underset{\left(0.0440\right)}{0.6057{\;}^{***}}$
S1	$\underset{\left(1.3078\right)}{11.9399{\;}^{***}}$	$\underset{\left(2.1234\right)}{-11.0163{\;}^{***}}$	$\underset{\left(3.1570\right)}{11.9912{\;}^{***}}$	$\underset{\left(0.0312)\right)}{-0.0490}$
S2	$\underset{\left(1.2800\right)}{5.5726{\;}^{***}}$	$\underset{\left(2.0783\right)}{-9.5696{\;}^{***}}$	$\underset{\left((3.0900)\right)}{9.7667{\;}^{***}}$	$\underset{\left(0.0306\right)}{-0.0202}$
S3	$\underset{\left(1.2811\right)}{14.2356{\;}^{***}}$	$\underset{\left(2.0801\right)}{-6.1080{\;}^{**}}$	$\underset{\left(3.0926\right)}{12.7661{\;}^{***}}$	$\underset{\left(0.0306\right)}{-0.0735{\;}^{**}}$
S4	$\underset{\left(1.2881\right)}{2.1922{\;}^{*}}$	$\underset{\left(2.0914\right)}{-7.6131{\;}^{***}}$	$\underset{\left(3.1094\right)}{5.9668{\;}^{*}}$	$\underset{\left(0.0308\right)}{0.0042}$
S5	$\underset{\left(1.2791\right)}{10.7613{\;}^{***}}$	$\underset{\left((2.0768\right)}{-10.7401{\;}^{***}}$	$\underset{\left(3.0877\right)}{6.8457{\;}^{**}}$	$\underset{\left(0.0305\right)}{-0.0209}$
S6	$\underset{\left(1.2813\right)}{5.4104{\;}^{***}}$	$\underset{\left(2.0804\right)}{-8.4761{\;}^{***}}$	$\underset{\left(3.0931\right)}{8.3448{\;}^{***}}$	$\underset{\left(0.0306\right)}{0.0002}$
S7	$\underset{\left(1.2791\right)}{3.7122{\;}^{***}}$	$\underset{\left(2.0768\right)}{-7.3248{\;}^{***}}$	$\underset{\left(3.0878\right)}{5.6735{\;}^{*}}$	$\underset{\left(0.0305\right)}{-0.0321}$
S8	$\underset{\left(1.2834\right)}{-7.40764{\;}^{***}}$	$\underset{\left(2.0838\right)}{-2.4897}$	$\underset{\left(3.0981\right)}{4.3521}$	$\underset{\left(0.0307\right)}{0.0105}$
S9	$\underset{\left(1.3870\right)}{20.0910{\;}^{***}}$	$\underset{\left(2.2520\right)}{-12.7784{\;}^{***}}$	$\underset{\left(3.3482\right)}{17.4210{\;}^{***}}$	$\underset{\left(0.0331\right)}{-0.1031{\;}^{***}}$
S10	$\underset{\left(1.2800\right)}{11.8102{\;}^{***}}$	$\underset{\left(2.0783\right)}{-7.2666{\;}^{***}}$	$\underset{\left(3.0900\right)}{10.8198{\;}^{***}}$	$\underset{\left(0.0306\right)}{-0.0278}$
S11	$\underset{\left(1.284\right)}{5.9073{\;}^{***}}$	$\underset{\left(2.0840\right)}{-10.3945{\;}^{***}}$	$\underset{\left(3.0984\right)}{8.6740{\;}^{***}}$	$\underset{\left(0.0307\right)}{-0.0396}$
time	$\underset{\left(0.0038\right)}{-0.0041}$	$\underset{\left(0.0062\right)}{-0.0227{\;}^{***}}$	$\underset{\left(0.0092\right)}{0.0294{\;}^{***}}$	$\underset{\left(0.0001\right)}{0.0002{\;}^{**}}$

Note: Standard deviation in parenthesis; ∗ p<0.1,∗∗ p<0.05,∗∗∗ p<0.01.
